# Mitigating neurodegenerative diseases: the protective influence of baicalin and baicalein through neuroinflammation regulation

**DOI:** 10.3389/fphar.2024.1425731

**Published:** 2024-12-02

**Authors:** Rui Yang, Ranran Wang, Ajing Xu, Jian Zhang, Jing Ma

**Affiliations:** Department of Clinical Pharmacy, Xinhua Hospital, Shanghai Jiaotong University School of Medicine, Shanghai, China

**Keywords:** baicalin, baicalein, neuroinflammation, microglia, neurodegenerative diseases

## Abstract

Neurodegenerative diseases (NDDs) represent a category of serious illnesses characterized by the progressive deterioration of neuronal structure and function. The exploration of natural compounds as potential therapeutic agents has gained increasing attention in recent years owing to their wide range of pharmacological activities and minimal side effects. Baicalin (BAI) and baicalein (BE), polyphenolic flavonoids, derived from the root of *Scutellaria baicalensis*, evidently show potential in treating NDDs. This review provides an overview of the current understanding of the roles of BAI and BE in alleviating neuroinflammation, a pivotal pathological process implicated in various NDDs. Studies conducted prior to clinical trials have shown that BAI and BE exert protective effects on the nervous system in different animal models of NDDs. Furthermore, mechanistic studies indicate that BAI and BE exert anti-inflammatory effects by inhibiting pro-inflammatory cytokines, suppressing microglial activation, and regulating microglial phenotypes. These effects are mediated through the modulation of inflammatory signaling cascades, including Toll-like receptor 4 (TLR4), mitogen-activated protein kinase (MAPK), amp-activated protein kinase (AMPK), NOD-like receptor thermal protein domain-associated protein 3 (NLRP3) inflammasome, and nuclear factor erythroid 2-related factor 2 (Nrf2)/hemoglobin oxygenase-1 (HO-1). Overall, BAI and BE exhibit promising potential as natural compounds with anti-inflammatory properties and offer innovative therapeutic approaches for managing NDDs.

## 1 Introduction

*Scutellaria baicalensis* (*S. baicalensis*), commonly known as Huangqin or Chinese skullcap, is widely distributed in northern, northwestern, and southwestern China (Shang et al., 2010). It has also been found in the Soviet Union, Mongolia, North Korea, and Japan. Baicalin (BAI; syn.baicalein7-O-β-D-glucuronic acid) and baicalein (BE; 5, 6, 7-trihydroxyflavone), the principal compounds derived from the roots of *S. baicalensis*, are polyphenolic substances and members of the flavone subclass of flavonoids (Gaire et al., 2014). BAI and its aglycone, BE, have attracted increasing attention from the pharmaceutical industry because of their remarkable biological activities. These two flavonoids share structural similarities and can be converted into each other during metabolism within the body (Liang et al., 2017). BAI, as a glucuronide form of BE, is hydrolyzed by glucuronidases in the intestines, liver, and other tissues, releasing baicalein. This process allows baicalein to exert its biological activities directly. Conversely, BE can be conjugated with glucuronic acid to form BAI through glucuronidation reactions, primarily occurring in the liver. Their pharmacological properties have garnered significant attention in recent years, leading to extensive research on their various therapeutic applications.

BAI is formed by combining BE with glucuronic acid. Due to its glucuronic acid component, BAI exhibits higher water solubility but has relatively poor ability to penetrate the intestinal epithelium, requiring specific transport mechanisms or enzymatic action to enter the bloodstream. In contrast, BE has smaller molecular size and higher lipid solubility, allowing it to more easily penetrate the intestinal epithelium and enter the bloodstream directly. It does not require specific transport mechanisms or enzymatic assistance, making it more efficient in crossing the intestinal barrier (Zhang et al., 2007; Li et al., 2011). BAI is moderately absorbed in the stomach, with limited absorption in the small intestine and colon. Conversely, BE is efficiently absorbed in the stomach and small intestine; however, its absorption in the colon is somewhat restricted. Owing to body dynamics, BE is more completely absorbed and converted back to BAI in the systemic circulation through conjugative reactions. Circulating BAI is expected to return to the gastrointestinal tract via biliary excretion (Taiming and Xuehua, 2006). BAI cannot directly cross the intestine but is hydrolyzed to BE by enzymes and bacteria (Akao et al., 2000; Day et al., 2003). BAI can cross the blood–brain barrier (BBB), quickly spread to the cerebrospinal fluid, and peak at a concentration of 344 μg/L in approximately 30 min after the intravenous administration of 24 mg/kg BAI. BAI has a tendency to build up in the striatum, thalamus, and hippocampus, which helps reinforce its positive impact on the central nervous system (CNS) (Zhang et al., 2006; Huang et al., 2008). Hence, the spread of BAI in the brain reinforces its healing properties on the central nervous system. Upon oral administration of BAI, the presence of both unchanged BAI and BE conjugates of glucuronide and sulfate is reportedly observed in the serum (Lai et al., 2003). BAI and BE are excreted via both the biliary and renal pathways (Akao et al., 2009).

BAI and BE exhibit diverse pharmacological effects, and one of the primary areas of research is their potent antioxidant activity. They can eliminate free radicals and decrease oxidative stress, which helps protect cells from damage and may alleviate various diseases linked to oxidative stress, such as heart problems, brain disorders, and cancer (Waisundara et al., 2009; Ma et al., 2021; Wang et al., 2021; Chen et al., 2022; Nie et al., 2023). Furthermore, BAI and BE exhibit anti-inflammatory effects by modulating various inflammatory mediators and signaling pathways. Their capacity to suppress the generation of pro-inflammatory cytokines enhances their effectiveness in managing inflammatory disorders, such as arthritis, inflammatory bowel disease, and asthma (Chen et al., 2014; Zhang et al., 2014; Dinda et al., 2017; Wang et al., 2021; Wen et al., 2023). Moreover, BAI and BE have been investigated for their neuroprotective properties. They may offer neuroprotection through mechanisms such as reducing neuronal apoptosis, suppressing neuroinflammation, and promoting neuronal regeneration (Yuan et al., 2020; Zhao et al., 2021; Huang et al., 2024; Song et al., 2024). The characteristics of BAI and its aglycone BE make them potential options for treating neurodegenerative diseases (NDDs), such as Alzheimer’s disease (AD), Parkinson’s disease (PD), and stroke.

## 2 Literature search strategy

To provide an overview of the research articles on BAI and BE in neurodegenerative diseases, we searched the databases PubMed, Web of Science, Embase, and Scopus. The search strategy used in PubMed was as follows: ((“Neurodegenerative Diseases” [MeSH] OR “Alzheimer Disease” [MeSH] OR “Parkinson Disease” [MeSH] OR “Huntington Disease” OR “Amyotrophic Lateral Sclerosis” OR “Multiple Sclerosis”) AND (“Neuroinflammation” [MeSH] OR “Brain Inflammation” OR “Microglia Activation”)) AND (“Baicalin” OR “Baicalein” OR “Flavonoids” OR “Scutellaria baicalensis”). The search strategy used in Web of Science was as follows: ((“Neurodegenerative Diseases” OR “Alzheimer Disease” OR “Parkinson Disease” OR “Huntington Disease” OR “Amyotrophic Lateral Sclerosis” OR “Multiple Sclerosis”) AND (“Neuroinflammation” OR “Brain Inflammation” OR “Microglia Activation”)) AND (“Baicalin” OR “Baicalein” OR “Flavonoids” OR “Scutellaria baicalensis”). The search strategy used in Embase was as follows: ((“Neurodegenerative Diseases” OR “Alzheimer Disease” OR “Parkinson Disease” OR “Huntington Disease” OR “Amyotrophic Lateral Sclerosis” OR “Multiple Sclerosis”) AND (“Neuroinflammation” OR “Brain Inflammation” OR “Microglia Activation”)) AND (“Baicalin” OR ‘Baicalein” OR “Scutellaria baicalensis”). The search strategy used in Scopus was as follows: (TITLE-ABS-KEY (“Neurodegenerative Diseases”) OR TITLE-ABS-KEY (“Alzheimer Disease”) OR TITLE-ABS-KEY (“Parkinson Disease”) OR TITLE-ABS-KEY (“Huntington Disease”) OR TITLE-ABS-KEY (“Amyotrophic Lateral Sclerosis”) OR TITLE-ABS-KEY (“Multiple Sclerosis”)) AND (TITLE-ABS-KEY (“Neuroinflammation”) OR TITLE-ABS-KEY (“Brain Inflammation”) OR TITLE-ABS-KEY (“Microglia Activation”)) AND (TITLE-ABS-KEY (“Baicalin”) OR TITLE-ABS-KEY (“Baicalein”) OR TITLE-ABS-KEY (“Scutellaria baicalensis”)).

## 3 NDDs and neuroinflammation

NDDs encompass a broad spectrum of neurological disorders characterized by various clinical and pathological hallmarks, affecting specific subsets of neurons within distinct regions of the CNS, mainly including AD, PD, multiple sclerosis (MS), amyotrophic lateral sclerosis (ALS), Huntington’s disease (HD), and multiple system atrophy (MSA). Although the pathogenic mechanisms of these diseases are different, such as different protein aggregates and genetic variations, they all share the common hallmark of chronic neuroinflammation (Mayne et al., 2020). Increasing evidence has demonstrated that neuroinflammation may not merely be a consequence of protein aggregation; rather, it may initiate the accumulation of aggregates at the earliest phase of the disease process (Sosna et al., 2018; Gao et al., 2023).

Microglia, considered macrophages of the CNS, play an important role in neuroinflammation. Resting microglia, also known as M0 microglia, maintain the homeostasis of their adjacent environment during active immune surveillance (Gao et al., 2023). Upon exogenous or endogenous stimuli, microglia are rapidly activated and shift to an activated phenotype, which is typically characterized by two phenotypic states: a classically activated M1 phenotype or an alternatively activated M2 phenotype. Generally, the M1 phenotype is associated with pro-inflammatory and neurotoxic responses, whereas the M2 phenotype predominantly possesses anti-inflammatory and neuroprotective functions (Woodburn et al., 2021). During aging, microglia tend to display a dominant M1-like phenotype associated with neurotoxic responses (Ward et al., 2015). In NDDs, endogenous pathological protein aggregation, neuronal damage, and microglial-related neuroinflammation are interconnected via a positive feedback loop.

In AD, microglia become persistently activated by the aggregation of β-amyloid peptide (Aβ) and subsequently transform into the M1 phenotype. They release a wide variety of pro-inflammatory and toxic productions, amplifying immune responses, leading to neurotoxicity (Meda et al., 2001), and increasing the secretion of Aβ fragments and the aggregation of soluble β-amyloid (Tan and Seshadri, 2010). In PD, excessive aggregation of α-synuclein (α-syn) can be released either directly from neurons or via exosomes, thereby activating microglia. Subsequently, activated microglia exacerbate the disease by releasing cytokines and chemokines that enhance α-syn pathogenicity, induce neuron death, and further enhance microglia activation (Reimer et al., 2018; Pajares et al., 2020). Similar to PD, MSA is also associated with the pathological protein α-syn. Its hallmark pathological characteristic is the presence of glial cytoplasmic inclusions (GCIs), rich in α-syn, within oligodendrocytes. Misfolded α-syn serves as a main trigger for microglial activation, which is believed to accelerate α-syn aggregation and promote the apoptosis of oligodendrocytes (Vieira et al., 2015). In MS, microglia are not the primary triggers but are influenced by a wider immune disturbance (Ransohoff, 2016). They adopt a pro-inflammatory behavior, such as antigen presentation, phagocytosis, and secretion of cytokines and chemokines, which play a central role in the pathogenesis of MS (Li and Barres, 2018; Voet et al., 2019).

Neuroinflammation also plays a vital role in the progression of other NDDs. In ALS, microglia can become overactivated due to the aggregation of TAR DNA-binding protein 43 and Cu/Zn superoxide dismutase 1 (SOD1), producing pro-inflammatory cytokines, which not only directly damage neurons but also exacerbate ALS (Zhao et al., 2015; Calió et al., 2020; Yu et al., 2020). There is a significant increase in activated microglia and pro-inflammatory cytokine levels in the brains of patients with HD and *in vivo* models (Tai et al., 2007; Sivandzade et al., 2019; Subhramanyam et al., 2019; Saba et al., 2022), which promote neuronal cell death by inducing apoptosis and reactive oxygen species (ROS)/reactive nitrogen species (RNS) production and complement activation, enhancing excitotoxicity and mitochondrial damage (Smith et al., 2012; Saba et al., 2022). In addition to that, chronic cerebral hypoperfusion (CCH) is the common underlying pathophysiological mechanism, which is a major contributor to cognitive decline and degenerative processes (Tian et al., 2022). A continuous decrease in cerebral blood flow causes cell death, and the subsequent release of cell debris will induce the neuroinflammation–immune cascade reaction (Ma et al., 2017). During this process, microglia could turn into an M1 phenotype that releases pro-inflammatory cytokines that further aggravate neuroinflammation and tissue damage, promoting the development of cognitive dysfunction and degeneration (Ma et al., 2017; Tian et al., 2022).

In summary, pathogenic protein aggregation, neuronal damage, and microglial-related neuroinflammation exhibit mutually reinforcing patterns that exacerbate neurodegeneration. Therefore, controlling neuroinflammation is a potential target for NDD therapy. Natural products that modulate neuroinflammation have attracted considerable attention in this field.

## 4 The effects of BAI and BE on neuroinflammation-related signaling pathways

**FIGURE 1:**
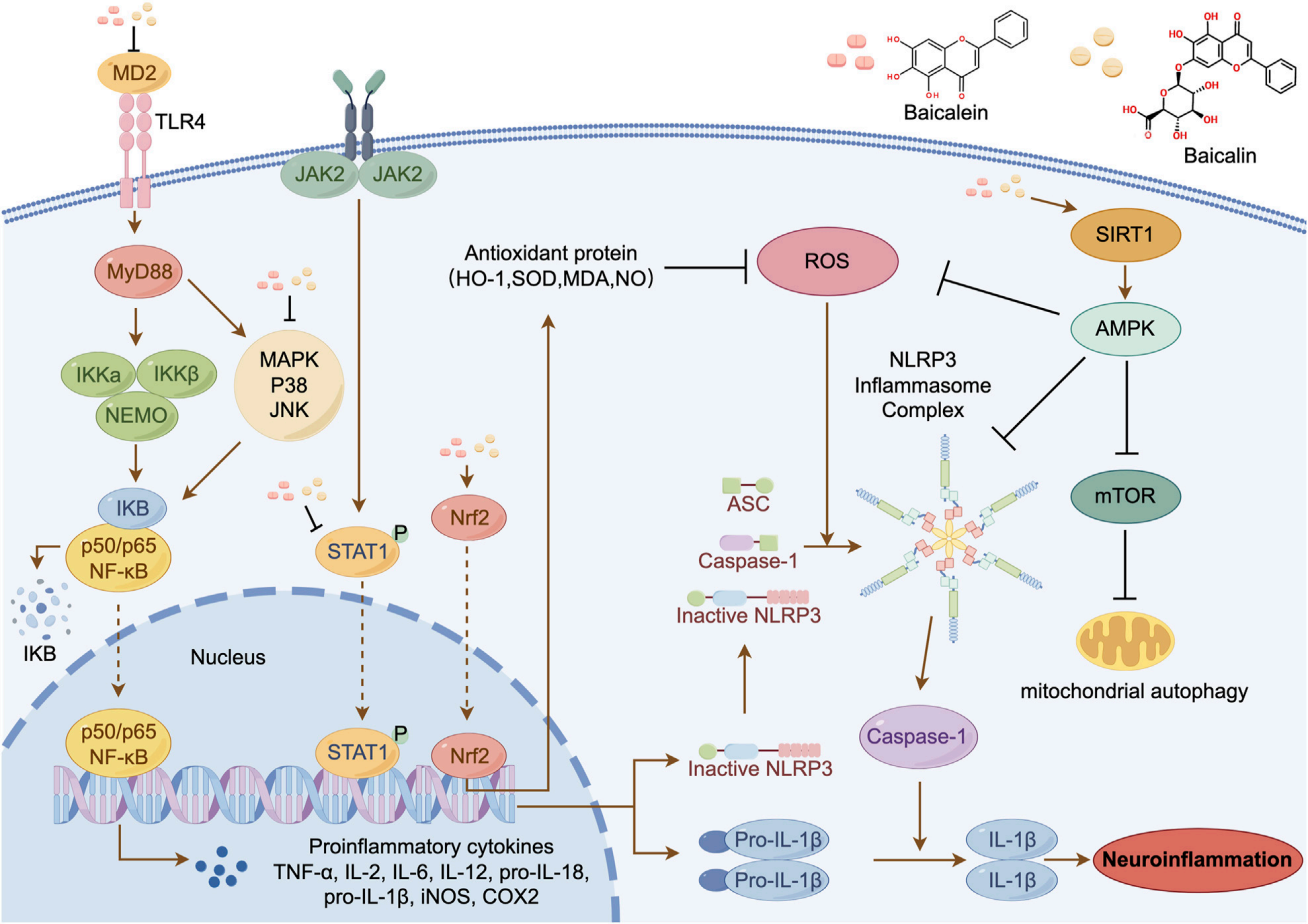
Plausible molecular mechanisms of BAI and BE in NDDs.

BAI and BE, essential natural flavonoids extracted from the roots of *S. baicalensis*, have various biological and pharmacological effects and can cross the BBB (Huang et al., 2008). Extensive research has demonstrated that BAI and BE can inhibit neuroinflammation by targeting various signaling pathways depending on specific NDDs (Figure 1) (Marogianni et al., 2020; Pan et al., 2021; Scheltens et al., 2021; Araujo et al., 2022; Zhang W. et al., 2023).

### 4.1 NF-κB signaling

Nuclear factor-kappa B (NF-κB), implicated in anti-inflammatory processes across various neurodegenerative conditions, typically resides in the cytoplasm bound to the regulatory protein inhibitor of NF-κB (IκB) (Sivandzade et al., 2019; Sun et al., 2022; Xu et al., 2024). When exposed to various stimuli, IκB gets phosphorylated by the IκB kinase complex, made up of IκB kinase (IKK) α, IKKβ, and the scaffolding protein NF-κB essential modulator, resulting in the release of NF-κB dimers. The dimers are subsequently transported into the nucleus and control the transcription of inflammatory cytokines like tumor necrosis factor α (TNF-α), interleukin-1 beta (IL-1β), interleukin (IL)-2, IL-6, IL-18, IL-12, inducible nitric oxide synthase (iNOS), and cyclooxygenase-2 (COX-2) (Guo et al., 2024). BAI or BE mediates anti-inflammatory effects by blocking NF-κB activation through multiple signal transductions such as Toll-like receptor 4 (TLR4), mitogen-activated protein kinase (MAPK), AMP-activated protein kinase (AMPK), and nuclear factor erythroid 2-related factor 2 (Nrf2)/heme oxygenase-1 (HO-1), ultimately exerting neuroprotective effects in NDDs.

### 4.2 TLR4 signaling

The TLR4 pathway has been implicated in neuroinflammation, which is a common manifestation of various CNS diseases (Adhikarla et al., 2021; Heidari et al., 2022). *In vitro* studies have shown that BAI inhibits microglial activation and the release of inflammatory factors induced by oxygen–glucose deprivation (OGD) or lipopolysaccharide (LPS). It additionally alters the regulation of associated proteins within the TLR4/MyD88/NF-κB signaling pathway. Interestingly, molecular docking analysis revealed that BAI binds favorably to the active site of TLR4-MD2, indicating a strong and stable interaction with the TLR4 receptor (Hou et al., 2012; Li B. et al., 2022). BAI inhibited neuroinflammation induced by microglia *in vivo* by blocking the TLR4/NF-κB pathway in amyloid beta precursor protein (APP)/presenilin-1 (PS1) mice (Jin et al., 2019). BAI triggered the TLR4/myeloid differentiation protein 88 (MyD88)/caspase-3 pathway to reduce neurodegeneration in the hippocampal CA3 area, while decreasing the levels of TLR4, NF-κB p65, iNOS, and COX-2 proteins and suppressing the secretion of TNF-α and IL-1β, which resulted in a protective effect on the nervous system (Tu et al., 2011; Yang et al., 2021). BE reduced the protein levels of TLR4, p-IκBα, and p-p65 in activated microglial models, hindering the translocation of NF-κB p65 from the cytoplasm to the nucleus and suppressing the expression of phosphorylated signal transducers and activators of transcription 1 (STAT1), which also contribute to the shift in microglial polarization toward an anti-inflammatory phenotype (Ran et al., 2021). The TLR4/MyD88/NF-κB pathway plays a role in CCH and is implicated in how BE prevents vascular dementia in rats (Song et al., 2024).

### 4.3 MAPK signaling

The MAPK signaling pathway plays a crucial role in controlling a variety of cellular functions such as cell proliferation, differentiation, and apoptosis. Emerging evidence has suggested a significant association between the dysregulation of the MAPK pathway and CNS disorders (Zheng et al., 2020; Tabaa et al., 2022; Khan et al., 2023). In the LPS-induced BV2 microglia model, Li et al. observed that BAI effectively inhibited LPS-induced phosphorylation of p38, extracellular signal-regulated kinase (ERK), and c-Jun N-terminal kinase (JNK), along with a reduction in the expression of neuroimmune mediators such as NO, prostaglandin E2 (PGE2), and IL-1β, suggesting that MAPK can be targeted by BAI (Li B. et al., 2022). In addition, BE treatment significantly inhibited p38, ERK 1/2, and JNK activation in the brains of PD rats, suggesting that BE can exert an anti-inflammatory effect in PD rats through the MAPK pathway (Zhang et al., 2017).

### 4.4 NLRP3 inflammasome signaling

The NOD-like receptor thermal protein domain-associated protein 3 (NLRP3) inflammasome, which includes NLRP3, apoptosis-associated speck-like protein containing a caspase recruitment domain (ASC), and caspase-1, breaks down inactive pro-IL-1β and pro-IL-18 to produce mature IL-1β and IL-18, respectively (Juliana et al., 2012; Yin et al., 2018). BAI decreased the activation of NLRP3 and production of IL-1β in the cortex of 3 × Tg-AD mice (Bitto et al., 2017), while BE decreased Aβ plaque accumulation and reduced NLRP3 inflammasome activation and neuronal cell death (Gong et al., 2023). The anti-inflammatory effect of BAI may be associated with its ability to inhibit the activation of the NLRP3 inflammasome, which occurs through the inhibition of NF-κB pathway activation—a preliminary step necessary for NLRP3 inflammasome activation (Zeng et al., 2017). Jin et al. demonstrated that BAI pretreatment significantly inhibited LPS/Aβ-induced elevation of p-IκBα expression and reduction in IκBα protein expression in BV2 microglial cells and simultaneously downregulated NLRP3 protein expression and inhibited caspase-1 activity, thereby reducing IL-1β and IL-18 levels (Jin et al., 2019). In PD, BAI showed a dose-dependent inhibition of the excessive phosphorylation of NF-κB p65 and the upregulation of NLRP3 inflammasomes, along with their resulting caspase-1 and IL-1β (Huang et al., 2024). In contrast, BE alleviated 1-methyl-4-phenyl-1,2,3,6-tetrahydropyridine (MPTP)-triggered neuroinflammation by inhibiting the NLRP3/caspase-1/gasdermin D (GSDMD) pathway (Rui et al., 2020). BAI treatment also enhanced the neuronal antioxidant capacity (Huang et al., 2021), which is associated with Nrf2/HO-1 activation (Ding et al., 2015; Li X. et al., 2022). The antioxidant effects mediated by Nrf2 are essential for the inhibition of NLRP3 inflammasome signaling by BE (Huang et al., 2024).

### 4.5 AMPK signaling

In various CNS disorders, the aberrant regulation of AMPK signaling has been implicated in the modulation of neuroinflammation (Chen et al., 2021a; Wang et al., 2022; Steinberg and Hardie, 2023; Wang et al., 2023). AMPK activation reduces the inflammatory response by blocking the NF-κB pathway (Jie et al., 2022). In PD, BE elevates AMPK phosphorylation and inhibits the mammalian target of rapamycin (mTOR) activity, whereas miR-30b-5p overexpression and sirtuin 1 (SIRT1) silencing partially abolish the function of BE in regulating the AMPK/mTOR pathway (Chen et al., 2021b). In addition, BAI attenuates neurological damage following OGD/reperfusion by inhibiting NLRP3 inflammasome activity through enhanced AMPK phosphorylation (Zheng et al., 2021; Li et al., 2017), while the inhibition of the AMPK/Nrf2 pathway may also contribute to BE’s neuroprotective mechanisms (Yuan et al., 2020)

### 4.6 Other possible mechanisms

The alteration of K (+)-Cl (−) co-transporter and Na-K-2Cl cotransporter-1 expression by BAI affects GABAergic signaling, while the enhancement of heat shock protein expression may also contribute to the neuroprotective mechanisms of BAI (Dai et al., 2013). The regulation of BE on microglial polarization is essential for suppressing neuroinflammation and nerve damage in AD by targeting the heme oxygenase 1/phosphodiesterase 4D axis (Gong et al., 2023).

## 5 The effects of BE and BAI on neuroinflammation in NDDs

Extensive research has indicated a close association between neuroinflammation and various neurodegenerative pathways (Marogianni et al., 2020; Scheltens et al., 2021; Araujo et al., 2022; Zhang W. et al., 2023). The neuroprotective properties of BAI and BE appear to stem from their anti-inflammatory characteristics (Pan et al., 2021) (Table 1).

**TABLE 1:** BAI and BE show protective effects on NDDs *in vivo* and *in vitro*.

Disease	Species	Models	Dose	Main findings	Reference
AD	1. C57BL/6 mice 2. BV2 cells	1. APP/PS1 AD model 2. Aβ/LPS	BAI: 103 mg/kg for 33 days 10, 20, and 40 µM into BV2 cells for 24 h	Inhibited microglia‐induced neuroinflammation and further attenuated cognitive function	Jin et al. (2019)
	C57BL/6 mice	3 × Tg-AD mice	BE: 100 mg/kg, 200 mg/kg, once per for 8 weeks	Improved the learning and memory ability by regulating the microglial phenotypic transformation and alleviating neuroinflammation	Xie et al. (2023)
	1. C57BL/6 mice 2. BV2 cells	1. APP/PS1 mice 2. LPS	BE: 100 mg/kg/d for 6 months 100 μM BAI for 24 h	Alleviated Alzheimer’s disease by promoting the microglial M2 polarization and reduced apoptosis	Gong et al. (2023)
	Rat	Aβ25-35	BE: 10, 50, and 100 µM into PC12 cells for 24 h	Reduced Aβ25–35-induced neuronal apoptosis and inflammation in PC12 cells	Ji et al. (2020)
	C57BL/6 mice	APP/PS1 mice	SQYZ: 5.55 g/kg, once per day for 60 days	Ameliorated the cognitive impairment and decreased the neural pathological changes in AD by regulating neuroinflammation, stress responses, and energy metabolism	An et al. (2018)
	BV2 cells	LPS	BAI: 2.5, 7.5, and 22.5 μM for 24 h	Inhibited LPS-stimulated neuroinflammation.	Li B. et al. (2022)
	BV2 cells	Aβ	BAI: 50 μM and 100 μM for 1 h and then with stimulators for 24 h	Inhibited Aβ-induced microglial overactivation *in vitro* and *in vivo*.	Xiong et al. (2014)
	C57BL/6 mice	Male J20 AD mice	BE: 80 mg/kg	Improved behavioral disorders and alleviated cerebral blood flow via reverting metabolic abnormalities	Zhang et al. (2023b)
PD	1. C57BL/6 mice 2. BV2 cells	1. MPTP 2. α-syn/MPP+	BAI: 50, 100, and 200 mg/kg 12.5, 25, and 50 µM into BV2 cells for 20 h	Showed protective effects in PD through the inhibition of microglial-related neuroinflammation and oxidative stress	Huang et al. (2024)
	*Caenorhabditis elegans*	6-OHDA	BAI: 1, 10, or 100 µM	Reduced 6-OHDA injury by suppressing apoptosis and oxidative stress	Ma et al. (2021b)
	1. C57BL/6 mice 2. Human cell line pLVXTet3G-α-syn SH-SY5Y	1. MPTP 2. Dox-induced	BAI: 20 and 40 mg/kg 12.5, 25, and 50 µM	Improved the PD model’s behavioral performance by the inactivation of neuroinflammation and oxidative stress	Lei et al. (2020)
	SD rats	6-OHDA	BAI: 50, 100, and 150 mg/kg	Showed a protective effect through an antioxidant, enhancing neurotransmitter release and regulating the metabolism of N-acetyl aspartate and glutamate	Tu et al. (2019)
	SD rats	6-OHDA	BAI: 25 mg/kg	Improved neuronal apoptosis	Zhai et al. (2019)
	PC12 cells	6-OHDA	BAI: 10, 20, 50, 70, or 100 µM	Reduced cell injury via downregulating miR-192-5p and regulation of PI3K/AKT and MDM-2/p53 signal pathways	Kang et al. (2019)
	Wistar rats	Rotenone	BAI: 78 mg/kg per day	Protected dopaminergic neuron damage by inhibiting iron accumulation in different brain regions.	Xiong et al. (2012)
	SH-SY5Y cells	Rotenone	BE: 10, 25, 50, or 100 µM	Showed neuroprotective effects by antioxidant activity, regulation of mitochondrial function, and anti- and pro-apoptotic proteins.	Song et al. (2012)
	Human wild-type α-syn	—	BE: 20 µM	Suppressed fibrillation of alpha-synuclein and enhanced disaggregation of existing fibrils	Xiong et al. (2012)
	C57BL/6 mice	Rotenone	BE: 300 mg/kg	Inhibited α-synuclein aggregation, decreased neuroinflammation, and maintained neurotransmitter homeostasis	Zhao et al. (2021)
	SD rats	Rotenone	BE: 200 and 400 mg/kg	Improved motor impairments by reducing brain damage and inhibiting the level of pro-inflammatory cytokine damage	Zhang et al. (2017)
	C57BL/6 mice	MPTP	BE: 1 mg/kg and 10 mg/kg	Attenuated motor ability and reduced DA neuron injury by inhibiting astroglia overactivation	Lee et al. (2014)
MS	C57BL/6 mice	MOG35-55	BE: 150 mg/kg	Delayed the onset of EAE and attenuated clinical symptoms, demyelination, and inflammatory cell infiltration	Ma et al. (2022)
	C57BL/6 mice	MOG35-55	BAI: 100 mg/kg/day	Reduced T-cell proliferation and affected the entire T-cell response	Zhang et al. (2015)
	SJL/J mice	Proteolipid protein (PLP) 139–151	BAI: 5–10 mg/kg	Inhibited a pre-activated immune system in the late effector phase, leading to disease eruption	Zeng et al. (2007)
	C57BL/6 male mice	Cuprizone	BE: 100 mg/kg	Attenuated motor dysfunction by reducing demyelination and neuroinflammation	Hashimoto et al. (2017)
	C57BL/6 mice	MOG35-55	BE: 25 mg/kg	Suppressed Th1 and Th17 differentiation *in vitro*; reduced the disease severity and infiltration process, attenuated demyelination in EAE, and blocked IL-17A production	Ying et al. (2023)
	C57BL/6 mice	MOG35-55	BAI: 75 mg/kg	Significantly attenuated the clinical severity of EAE by the inhibition of 12/15-LO and then inhibited microglia activation.	Xu et al. (2013)
CCH	SD rats	Bilateral common carotid artery occlusion (BCCAO)	BAI: 50 and 100 mg/kg	Ameliorated cognitive impairment in CCH-induced VD rats through its pro-remyelination and anti-inflammatory capacities, possibly by activating Wnt/β-catenin and suppressing NF-κB signaling	Xiao et al. (2023)
	Wistar rats	BCCAO	BE: 30 and 100 mg/kg	Have therapeutic potential for the treatment of dementia caused by CCH and contributed to its protections on brain mitochondrial homeostasis and function	He et al. (2009)
	SD rats	BCCAO	BE: 50 and 100 mg/kg	Ameliorated cognitive impairment, attenuated hippocampal inflammatory responses, inhibited the TLR4/MyD88/NF-κB signaling pathway, and modulated intestinal microbiota in VD rats	Song et al. (2024)
	SD rats	BCCAO	BE: 2.4 mg/kg/day	Improved the cognitive deficits and neuropathological changes induced by CCH in rats to its antioxidant action	Liu et al. (2007)
	SD rats	BCCAO	—	HRT (BI is one of the compounds) exerts anti-inflammatory effects via inhibition of p38 MAPK phosphorylation in the hippocampus of BCCAO rats	Sohn et al. (2019)
ALS	Buffalo heart cystatin	In the presence of glyoxal	BAI: 10–100 µM	Showed concentration-dependent anti-aggregation effects.	Sohail et al. (2018)
	Wild-type SOD1 or mutant A4V SOD1	DTT (80 mM) and EDTA (2 mM)	BAI: 5, 15, 30, and 90 µM	Showed potent antiamyloidogenic and fibril-destabilizing effects for Sod1 fibrils.	Bhatia et al. (2020)
	G93A/SOD1 mouse	G93A/SOD1 mouse	BE: 3.7 or 7.4 nmol/kg/day	Downregulated inflammation-related gene expression	Ignacio et al. (2005)
HD	Wistar rats	Quinolinic acid (QA) intrastriatal administration	BE: 10 and 30 mg/kg	Restored the level of enzymatic and non-enzymatic antioxidants and mitochondrial complexes by reducing the levels of inflammatory mediators and maintaining the level of BDNF and GDNF	Purushothaman and Sumathi (2022)
MSA	OLN-93 oligodendrocyte cell line	OLN-t40 and OLN-AS cells	BE: 100 µM	Inhibited fibrillation of α-syn *in vitro*	Kragh et al. (2009)

### 5.1 BAI, BE, and AD

AD is the most common neurodegenerative disorder, characterized by significant pathological changes in the brain, including the accumulation of Aβ plaques and neurofibrillary tangles formed hyperphosphorylated tau protein (Scheltens et al., 2021; Thakur et al., 2023). The presence of Aβ plaques and tau tangles leads to chronic neuroinflammation, which exacerbates neuronal damage and cognitive decline (Leng and Edison, 2021). TLR4, NF-κB, MAPK, and the NLRP3 inflammasome are key components in triggering microglial-related neuroinflammation. TLR4 facilitates the recognition of pathological stimuli and then initiates NF-κB and MAPK pathways, which propagate pro-inflammatory signaling (Wu et al., 2022). The NLRP3 inflammasome can be activated by the TLR4/MyD88/NF-κB signaling pathway or directly by oligomers and fiber Aβ, amplifying cytokine release and perpetuating a cycle of neuroinflammation (Yang et al., 2020; Milner et al., 2021). Targeting the inflammation-related signaling pathway could suppress microglial activation, reduce pathogenesis, and improve learning and memory functions, which may be effective therapeutic strategies for AD.

Both BAI and BE exhibit beneficial effects in AD by modulating neuroinflammation. In an Aβ-induced cell model, BAI attenuates Aβ-mediated microglial inflammatory responses and neuroinflammation‐associated neuronal apoptosis (Jin et al., 2019; Xiong et al., 2014). Similar effects have been observed with BE, which can alleviate Aβ25–35-stimulated neuronal apoptosis and inflammation (Ji et al., 2020). In addition, BAI significantly alleviates LPS-induced neuroinflammation by suppressing the expression of miR-155, regulating the TLR4/MyD88/NF-κB pathway and MAPK pathway (Li B. et al., 2022).

Furthermore, BAI and BE have demonstrated notable neuroprotective effects in various animal models of AD. BAI treatment attenuated spatial memory dysfunction in APP/PS1 mice by suppressing microglial overactivation (Jin et al., 2019). In a J20 APPSwInd transgenic (Tg) mouse model of AD, BE inhibited hyperactivity and improved spatial learning ability. Metabolic profiling of specific brain regions indicated that BE regulates neuroinflammation, which is associated with the modulation of starch, sucrose, and glycolipid metabolism in the cortex and hippocampus (Zhang L. et al., 2023). Additionally, SQYZ granules, a Chinese herbal preparation containing BAI, ameliorated neural pathological changes in AD, possibly by modulating anti-neuroinflammation, promoting stress recovery, and enhancing energy metabolism in APP/PS1 mice (An et al., 2018).

Moreover, BE and BAI mitigated neuroinflammation by modulating the phenotypic transformation of activated microglia. BE improved cognitive dysfunction in 3 × Tg-AD mice by enhancing M2 microglial polarization (Xie et al., 2023). Furthermore, in both APP/PS1 double-transgenic mice and LPS-stimulated BV2 microglia, BE significantly shifts the microglial phenotype from M1 to M2 (Gong et al., 2023). Moreover, in LPS/interferon γ-induced neuroinflammation, BAI promoted the polarization of microglia from the M1 phenotype to M2 phenotype (He et al., 2022). In conclusion, these studies clearly demonstrated that the protective effects of BAI and BE against neuronal damage in AD are related to the inhibition of neuroinflammation.

### 5.2 BAI, BE, and PD

PD is a complex neurodegenerative disorder characterized by several pathological manifestations, including the aggregation of α-synuclein, loss of dopaminergic neurons in the nigrostriatal system, and heightened neuroinflammation. The presence of protein aggregates and damaged neurons in PD can initiate an inflammatory response (Gao et al., 2024). Activated microglia release pro-inflammatory cytokines such as IL-1β, IL-6, and TNF-α. These cytokines can further damage neurons and exacerbate the disease process through autocrine and paracrine mechanisms. Additionally, neuroinflammation and oxidative stress can trigger the activation of JNK and p38 MAPK pathways, both of which are involved in exacerbating the pathological progression of PD (Gravandi et al., 2023; Gogna et al., 2024). Currently, dopamine replacement therapy remains the primary treatment approach for PD. However, it is important to note that although these methods are widely used, they may not be universally effective or suitable for all patients.

In a previous investigation using a *Caenorhabditis elegans* model of PD, it was discovered that BAI enhanced reversal and omega turn behavioral characteristics, along with increasing survival rates. BAI provided protection against 6-hydroxydopamine (6-OHDA) damage by preventing cell death and decreasing oxidative damage (Ma J. et al., 2021). Other *in vitro* experiments have demonstrated the neuroprotective effects of BAI in PD. BAI inhibited the inflammatory response caused by α-syn/1-methyl-4-phenylpyridinium (MPP+) in BV2 cells (Huang et al., 2024) and reduced neurotoxicity induced by 6-OHDA in PC12 cells (Kang et al., 2019). Additionally, BAI and BE reportedly increase the viability and reduces cell death in dopaminergic SH-SY5Y cells (Song et al., 2012; Lei et al., 2020). Numerous animal models have confirmed the neuroprotective effects of BAI against PD. BAI demonstrated a notable defense mechanism in PD rats induced by 6-OHDA, possibly through antioxidant actions, enhanced neurotransmitter release, and control of N-acetyl aspartate and glutamate metabolism. BAI attenuated substantia nigra neuronal apoptosis in PD rats (Tu et al., 2019; Zhai et al., 2019). Moreover, BAI demonstrates neuroprotective effects in MPTP-induced PD mice by inhibiting pro-inflammatory cytokine expression and reducing oxidative stress. BE also has exhibited a strong protective effect against PD (Lei et al., 2020; Huang et al., 2024). In PD, BE has the potential to modulate pathways mediated by α-syn beyond directly targeting α-syn itself (Li et al., 2023). BE attenuates iron accumulation and iron-induced oxidative stress in the brain of PD rats (Liu et al., 2022). BE has the potential to interfere with both wild-type and E46K/H50Q mutant α-syn fibrils, potentially mitigating PD progression (Yao et al., 2022). Treatment with BE decreases the accumulation of α-syn, prevents inflammation in the brain, balances neurotransmitter levels, and decreases the release of inflammatory molecules like TNF-α and IL-6, all while regulating the activity of astrocytes and microglia in rats with PD (Lee et al., 2014; Zhang et al., 2017; Zhao et al., 2021). These results indicate that BAI and BE have beneficial therapeutic effects in PD, possibly attributed to their anti-inflammatory, antioxidant, and other related actions.

### 5.3 BAI, BE, and MS

MS is a chronic autoimmune demyelinating disease affecting the central nervous system. Several cytokines play crucial roles in MS, with interferon-gamma (IFN-γ) being produced by activated T cells. IFN-γ binds to its receptor on various central nervous system cells, including astrocytes and microglia, activating the Janus kinase (JAK)–STAT signaling pathway (Kooshki et al., 2023). The associated JAKs are phosphorylated upon receptor activation, which, in turn, phosphorylates STAT proteins. These STAT proteins then dimerize and translocate to the nucleus, regulating the expression of genes related to immune responses and inflammation (Benucci et al., 2023). Additionally, TNF-α plays a significant role by binding to its receptors and activating multiple intracellular pathways, including the NF-κB pathway, which further enhances the expression of pro-inflammatory genes.

Studies of MS have demonstrated that BAI reduces the entry of immune cells into the CNS, suppresses the production of inflammatory substances and chemokines, and hinders the formation of Th1 and Th17 cells (Zhang et al., 2015). Furthermore, BAI can inhibit the development and progression of experimental autoimmune encephalomyelitis (EAE), which is an animal model that has a significant relationship with MS (Zeng et al., 2007). The demyelination in EAE is caused by an autoimmune response that is artificially induced and shares similarities with the unknown trigger of MS demyelination (Manogaran et al., 2018). BE also blocks the activation of microglia/macrophages toward the M1 phenotype in EAE mice by focusing on STAT 1 (Ma et al., 2022) and reduces cuprizone-induced demyelination by suppressing neuroinflammation (Hashimoto et al., 2017). Additional research has indicated that BE can improve EAE by reducing pathogenic C-X-C motif chemokine receptor 6 (CXCR6)+ CD4 cells (Ying et al., 2023) or blocking 12/15-lipoxygenase (Xu et al., 2013). These findings emphasize the importance of exploring the potential roles of BAI and BE in improving therapeutic strategies for MS.

### 5.4 BAI, BE, and CCH

CCH is characterized by a persistent reduction in cerebral blood flow, which often develops gradually and can significantly impact brain function. This condition activates the MAPK pathway, involving various members of the MAPK family, including ERK, JNK, and p38 MAPK (Zhang et al., 2020). The activation of these kinases is triggered by stimuli associated with the ischemic environment, such as oxidative stress and inflammatory cytokines. In the context of chronic cerebral ischemia, NF-κB is also activated. ROS generated during ischemia, along with other inflammatory mediators, stimulate the activation of the IKK complex (Su et al., 2017).

In addition, numerous studies have demonstrated that CCH is a major contributor to neurodegenerative processes (Daulatzai, 2017). Recent studies have shown that BAI exerts neuroprotective effects against chronic brain ischemia. BAI ameliorates cognitive impairment in CCH-induced VD rats through its pro-remyelination and anti-inflammatory abilities, possibly by activating Wnt/β-catenin and suppressing NF-κB signaling (Xiao et al., 2023). BE alleviates cognitive and motor impairments by decreasing mitochondria reactive oxygen species production (He et al., 2009) and inhibiting the TLR4/MyD88/NF-κB signaling pathway in CCH rats (Song et al., 2024; Liu et al., 2007). Traditional Chinese medicine—Hwangryunhaedok-Tang—containing BAI and BE improves cholinergic dysfunction and inhibits neuroinflammatory responses in CCH rats (Sohn et al., 2019). These results indicate that BAI has a beneficial therapeutic effect against CCH-induced brain injury through its anti-inflammatory properties.

### 5.5 BAI, BE, and other NDDs

ALS is a severe and progressive neurodegenerative disorder primarily affecting the motor neurons in the brain and spinal cord (Manogaran et al., 2018). HD is an inherited, progressive neurodegenerative disorder that significantly impacts both the physical and mental health of those affected (Gharaba et al., 2024). MSA is a rare and progressive neurodegenerative disorder that impacts multiple systems in the body (Ndayisaba et al., 2024). The MAPK and NF-κB signaling pathways play crucial roles in the pathophysiology of these NDDs.

BAI exhibits concentration-dependent anti-aggregation effects linked to ALS (Sohail et al., 2018). In ALS mouse models, the impact on mutant SOD1 was more pronounced than that on wild-type SOD1, affecting fibril elongation (Bhatia et al., 2020; Ignacio et al., 2005). BE also mitigates the psychological and behavioral changes induced by quinolinic acid in an HD mouse model (Purushothaman and Sumathi, 2022). BE effectively decreases the number of cells exhibiting microtubular retraction and suppresses the aggregation of α-syn in the MSA model (Kragh et al., 2009). In conclusion, BAI and BE demonstrate the potential to ameliorate NDD-associated symptoms.

## 6 Conclusion and future perspective

NDDs have a profound impact on human health, primarily characterized by the progressive loss of neuronal function and structure, leading to cognitive decline and physical disabilities. Current therapeutic strategies for these disorders are limited, with most treatments focusing on alleviating symptoms rather than addressing the underlying pathology.

Extensive evidence has demonstrated the potent anti-inflammatory and neuroprotective properties of BE and BAI in both *in vitro* and *in vivo* models of various NDDs mediated through the activation of multiple signaling pathways. In recent years, two phase-I clinical trials involving BE chewable tablets were completed in healthy Chinese adults, demonstrating that oral BE administration was safe and well-tolerated in healthy subjects (Pang et al., 2016). In a randomized, double-blind, placebo-controlled trial of BAI in patients with coronary heart disease (CAD) and rheumatoid arthritis (RA), it was found that BAI reduces blood lipids and inflammation in patients with both CAD and RA (Hang et al., 2018). BAI affects the innate immunity and apoptosis in leukocytes of children with acute lymphocytic leukemia (Orzechowska et al., 2014). A clinical study on BAI in patients with ulcerative colitis (UC) demonstrated that BAI can balance immune function and alleviate inflammation associated with UC (Yu et al., 2015). Additionally, several clinical trials have investigated BAI or herbal formulations containing BAI in patients with mild–moderate photo-damaged skin, non-surgical periodontal therapy, or after the surgical removal of impacted mandibular third molars (Farris et al., 2014; Isola et al., 2021; Isola et al., 2019). However, there remains a lack of targeted clinical research specifically investigating the effects of BAI and BE on NDDs.

To fully explore the therapeutic potential of BAI and BE, future clinical trials should focus on assessing their efficacy in patients with neurodegenerative disorders. Furthermore, additional research is required to characterize their specific molecular targets or receptors, which is essential for understanding their pharmacological mechanisms, optimizing drug design, and advancing drug development. Given the limited water and lipid solubility of BAI and BE, developing innovative delivery systems—such as nanoparticles or other advanced carriers—will be crucial to enhance bioactivity, improve blood–brain barrier permeability, and increase clinical efficacy (Zhou et al., 2017).

Despite these challenges, accumulating evidence highlights the value of BAI and BE as promising natural compounds. Their therapeutic potential merits continued investigation and exploration, offering hope for new treatment options in the medical and healthcare fields.
